# Supplementary material for: Emergence of resistance to last-resort antimicrobials in bacteremia patients: A multicenter analysis of bloodstream pathogens in Korea
Source: PLoS One. 2024 Oct 23;19(10):e0309969. doi: 10.1371/journal.pone.0309969 (PMC11498668; doi:10.1371/journal.pone.0309969)
Supplement: S1 Table — (DOCX) [file pone.0309969.s001.docx]

**S1 Table.** Prevalence of microbial isolates in blood cultures across three hospital settings

| **Hospital S (n=905)** | | **Hospital A (n=2,070)** | | **Hospital P (n=422)** | |
| --- | --- | --- | --- | --- | --- |
| Isolates | N | Isolates | N | Isolates | N |
| *Escherichia coli* | 296 | *Escherichia coli* | 453 | *Staphylococcus epidermidis* | 74 |
| *Klebsiella pneumoniae* | 114 | *Staphylococcus epidermidis* | 200 | *Enterococcus faecium* | 34 |
| *Staphylococcus epidermidis* | 81 | *Klebsiella pneumoniae* | 169 | *Acinetobacter baumannii* | 33 |
| *Staphylococcus aureus* | 61 | *Staphylococcus aureus* | 143 | *Staphylococcus capitis* | 31 |
| *Staphylococcus capitis* | 47 | *Enterococcus faecium* | 83 | *Escherichia coli* | 28 |
| *Staphylococcus hominis* | 44 | *Staphylococcus capitis* | 66 | *Staphylococcus aureus* | 24 |
| *Proteus mirabilis* | 20 | *Bacillus* species | 64 | *Klebsiella pneumoniae* | 20 |
| *Enterococcus faecium* | 18 | *Enterococcus faecalis* | 56 | *Corynebacterium striatum* | 19 |
| *Staphylococcus caprae* | 14 | *Pseudomonas aeruginosa* | 45 | *Enterococcus faecalis* | 17 |
| *Staphylococcus pettenkoferi* | 14 | *Staphylococcus hominis* | 40 | *Staphylococcus hominis* | 17 |
| *Staphylococcus haemolyticus* | 13 | Coagulase Negative *Staphylococcus* | 37 | *Candida albicans* | 15 |
| *Streptococcus agalactiae* | 13 | *Enterobacter cloacae* | 34 | *Staphylococcus haemolyticus* | 13 |
| *Enterococcus faecalis* | 12 | *Staphylococcus caprae* | 31 | *Staphylococcus caprae* | 12 |
| *Acinetobacter baumannii* | 11 | *Candida albicans* | 31 | *Staphylococcus hominis* | 11 |
| *Pseudomonas aeruginosa* | 11 | *Corynebacterium striatum* | 27 | *Staphylococcus warneri* | 8 |
| *Klebsiella oxytoca* | 8 | *Proteus mirabilis* | 22 | *Candida glabrata* | 4 |
| *Candida albicans* | 7 | *Staphylococcus haemolyticus* | 21 | *Proteus mirabilis* | 4 |
| *Klebsiella variicola* | 6 | *Streptococcus mitis* group | 20 | *Staphylococcus pettenkoferi* | 3 |
| *Streptococcus constellatus* | 6 | *Enterobacter aerogenes* | 19 | *Streptococcus mitis/Streptococcus oralis* | 3 |
| *Streptococcus salivarius* | 6 | *Acinetobacter baumannii* | 18 | *Enterobacter cloacae* | 3 |
