# Supplementary material for: Emergence of resistance to last-resort antimicrobials in bacteremia patients: A multicenter analysis of bloodstream pathogens in Korea
Source: PLoS One. 2024 Oct 23;19(10):e0309969. doi: 10.1371/journal.pone.0309969 (PMC11498668; doi:10.1371/journal.pone.0309969)
Supplement: Attachment — Submitted filename: Emergence of resistance to last-resort antimicrobials.docx [file pone.0309969.s002.docx]

**REVIEWER’S COMMENTS**

**Title: Emergence of resistance to last-resort antimicrobials in bacteremia pathogens: A multicenter analysis of bloodstream pathogens in Korea**

**Summary**

This is an important research work which brings to fore the profile of AMR associated with blood stream infection in Seoul, South Korea. It is a retrospective multi-centre study conducted within a year period across three health facilities (two secondary and one tertiary health facility) with the aim of conducting a large-scale analysis that enables adequate review of the microbiological and clinical characteristics of patients diagnosed with bacteremia. The objectives of the study and rationale for the study were well highlighted. The problem statements and gap in knowledge were also well outlined. Reference to previous literature were satisfactory.

The authors described their methods broadly which is worrisome for reproducibility. To summarize, the authors reviewed positive blood culture results to identify bacteria isolates, conducted antimicrobial susceptibility testing and compared the pattern of empirical treatment and tailored treatments with respect to their spectrum of coverage and MDRO. They went further to assess the underlying pathology and in-hospital mortality of the patients. However, there are important questions to answer. The authors conducted this study in only urban hospitals which have implications for generalizability to a more heterogenous population. The study sites should be sufficiently described to put the study in context. The sampling method and study population were not adequately described. The study population appears to be skewed to the elderly based on the median age group despite the fact that bacteremia can occurs in any age group. It is important that the authors clearly outline the inclusion and exclusion criteria of the study population.

Although, the variables of interest were well elucidated, there was no mention of the study instrument which has implications for standardization being a multi-centre study. Were the samples analyzed separately in each facility or pooled to a centre and were there clear Laboratory protocols developed? What were the other quality assurance processes for the laboratory component of this study?

The research findings provide evidence of antimicrobial resistance to last resort antibiotics. Analysis done are mostly descriptive, authors should consider doing some inferential statistics or if done to be reflected in the tables. In addition, given the comparative nature of the study with respect to facilities, statistically significant differences should also be highlighted (asterix) on the tables where available. It is also important that the authors explain how 3397 bacteria isolates were isolated from 3064 blood patients, having mentioned in the methods, that they focused solely on the first organism isolated from each positive blood culture per patient. Figure 1, should add up to 100% for each health facility (showcasing MDROs and non-MDROs even if the MDROs are disaggregated). The variables in figure 2 are best presented using a table to compare the ASC scores between Empirical and Tailored treatment and highlighting the level of statistical significance.

The authors may also wish to review the manuscript for typographical and grammatical errors beginning with the abstract, line 27-28. Also, line 50-57 (The sentence is too long, authors to consider splitting into 2 or 3 sentences). Rephrase sentence in line 77-80. The discussion in line 177-183 is a bit confusing.

Overall, the study findings highlight the need for rapid diagnostic infrastructure with capacity for wide antimicrobial resistance detection in order to reduce the use of empirical antibiotics and enhance timely prescription of tailored treatment which is critical to slow down the emergence of multidrug resistant organisms.

I personally think this is a well written manuscript, the findings are noteworthy and should be used to drive a strong public health response in South Korea to address the issues of antimicrobial resistance. I recommend that the manuscript be published after the outlined corrections are effected.
